# Horizontal Palpebral Fissure Best Predicts Subjective Facial Asymmetry in Unilateral Anophthalmia/Microphthalmia

**DOI:** 10.1097/IOP.0000000000003063

**Published:** 2025-10-21

**Authors:** Emiel J. Romein, Annabel L. W. Groot, Jelmer S. Remmers, Pim de Graaf, Niels P. T. J. Liberton, Birgit I. Lissenberg-Witte, Annette C. Moll, Peerooz Saeed, Dyonne T. Hartong

**Affiliations:** * Department of Ophthalmology, Amsterdam Orbital Center, Amsterdam UMC, University of Amsterdam; † Department of Radiology and Nuclear Medicine; ‡ Department of Ophthalmology, Cancer Center Amsterdam; § Department of Medical Technology, 3D Innovation Lab; ‖ Department Epidemiology and Data Science, Amsterdam UMC, Vrije Universiteit Amsterdam, Amsterdam, Netherlands

## Abstract

**Purpose:**

Congenital anophthalmia/microphthalmia are developmental eye disorders with variable severity. The absence of a normal-sized eye can cause facial asymmetry. Outcome is often reported as relative horizontal palpebral fissure (rHPF) and/or orbital dimensions. Yet, the predictive value of these measurements is unknown. This study aims to test the relation between facial and ultrasound measurements and subjective outcomes.

**Methods:**

In this retrospective study, 3D facial scans of 31 patients with unilateral an/microphthalmia were analyzed. The scans were obtained using the Vectra scanner, and measurements were taken using GOM Inspect software. Various facial landmarks were identified, including horizontal palpebral fissure (HPF), lower lid, upper eyelid, lid crease, and inferior eyebrow margin. Orbital width, orbital height, and axial eye length were measured using ultrasonography. Ratios between the affected and unaffected sides were calculated from these measurements. Faces were also subjectively rated for the degree of asymmetry on a scale of 1 to 5.

**Results:**

Average subjective judgment showed a significant association with, respectively, rHPF (*p* < 0.001), caudal placement of the eye (*p* < 0.001), orbital width (*p* = 0.001), pretarsal show asymmetry (*p* = 0.003), and axial length (*p* = 0.003). After a forward selection procedure, only rHPF predicted the subjective outcome. rHPF was strongly correlated with the other significant factors.

**Conclusion:**

The results indicate that the rHPF is best associated with subjective outcome. The forward selection procedure showed that adding ultrasound or other facial measurements did not result in a better association.

## Abstract 2

Horizontal eyelid symmetry is the best predictor for subjective outcome in anophthalmic and microphthalmic patients and can be used as the main outcome parameter in clinical practice and in studies evaluating the effect of treatments.

Congenital microphthalmia/anophthalmia (MICA) are rare developmental deficits of the eyeball in fetal development. This results in a wide range of phenotypes from subtle developmental disorders with a mild decrease in eye size, to disorders where the eye is extremely small or even totally absent.^1–3^

The absence of a normal-sized eye is associated with a growth deficiency of orbital structures and soft tissue around the eye.^1^ Disproportionate growth of the affected orbit and eyelids results in various asymmetries, which may impact quality of life.^3–5^ Parents of these newborn babies are often looking for treatment of the already visible abnormality and/or to prevent the development of future increasing facial asymmetry.

Treatment to expand the eye socket is generally done using conformers, starting as early as possible.^6–10^ The goal is to stimulate growth of the eye socket by periodic increase of the conformer size. Others have inserted inflatable silicone implants (directly in the socket or intraorbital through a lateral canthotomy with tunneled injection tube or a rim-fixed inflation device) or self-expanding spheres/hemispheres or pellets into the orbit with the aim to have a direct effect on orbital bone.^10–17^

However, there is no standard to determine a good clinical outcome. Earlier studies used the relative horizontal palpebral fissure (rHPF) width and orbital dimensions as objective clinical outcomes.^18–20^ The golden standard to measure orbital dimensions is with CT. Yet, this measurement is rather avoided in children due to harmful radiation. In our previous study, we have been able to measure these orbital rim dimensions with ultrasound measurements.^18^ It is, however, not known whether the bony orbital dimensions are directly translated to the clinical outcome. In addition, three-dimensional scans have been shown to measure facial deformities in a standardized manner using predetermined facial landmarks.^21^

The aim of this study is to elaborate whether 3D scanned facial measurements using predetermined landmarks and bony rim measurements performed with ultrasonography, relate to subjective judgment of the affected facial area. We will also include axial length (AL) measurements to gain insight into the overall effect of the treatment itself.

We will test multiple facial and orbital dimensions to gain insight into what factors most accurately predict the subjective judgment. Which parameters, facial or orbital, are best used to reflect clinical outcome in microphthalmic and anophthalmic patients?

## METHODS

### Study Design and Study Population

For this retrospective study, we used the population of MICA patients receiving ultrasonography in combination with a 3D facial scan in the Amsterdam University Medical Center, Amsterdam, the Netherlands. The data were captured from 2015 to 2022. All patients underwent 3D facial scans and ultrasonography as part of their standard follow-up care. Only cases in which the facial scan and ultrasonography were performed on the same day or several days apart were included. The facial scans had to be of good quality with the patient in an upright position. Both anophthalmic and microphthalmic unilateral patients could be included, as well as patients with intraorbital cysts. All ages, treatment types, or stages of treatment could be included since it did not influence our study question, except that babies often did not have a good quality set of scans. Bilateral cases were excluded since those cases have a more complex comparison. During the 3D scan, patients wore their ocular prosthesis. This study got ethical approval by the Ethics Committee of the Amsterdam University Medical Center. Parents and (where possible) patients gave informed consent for the use of the scans for scientific use. This study was performed according to the tenets of the Declaration of Helsinki.

### 3D Scanning

We three-dimensionally scanned our patients using the Vectra M3-2, version 5.7.2 scanner. The scanner uses 3 separate cameras to combine it into a single 3d file (OBJ). The children were positioned in the right position and, if needed, supported by their parents.

### 3D Measurements

The OBJ models were uploaded into GOM Inspect 2020 (GOM GmbH, Braunschweig, Germany) to construct 3D measurements. In GOM, we placed the OBJ models into a coordinate system where the nose bridge was centered on the origin (0,0,0 coordinate). The nasal and lateral canthi were manually marked, and the HPF line was drawn in between the medial and lateral canthus. On this line, points were set at 0%, 25%, 50%, 75%, and 100% of the distance from nasal to lateral canthus. Perpendicular lines were drawn through these points to set landmarks at the lower eyelid, upper eyelid (UL), lid crease, and inferior eyebrow margin, see Figure. The lid crease was identified as the fold on the UL. In some patients, where lid crease was absent, this was noted as absent. From these points, we obtained the following measurements: HPF length (distance between medial and lateral canthus), vertical palpebral fissure length (VPF, distance from central lower eyelid to central UL), pretarsal show (length from the UL to the lid crease), and brow height (from the UL to the inferior eyebrow margin). We also determined the contour of the eyelids by making a ratio of the HPF (rHPF) and VPF (rVPF). To judge symmetry, we used relative values by dividing the affected eye measurement by the unaffected eye measurement.

**FIG.**
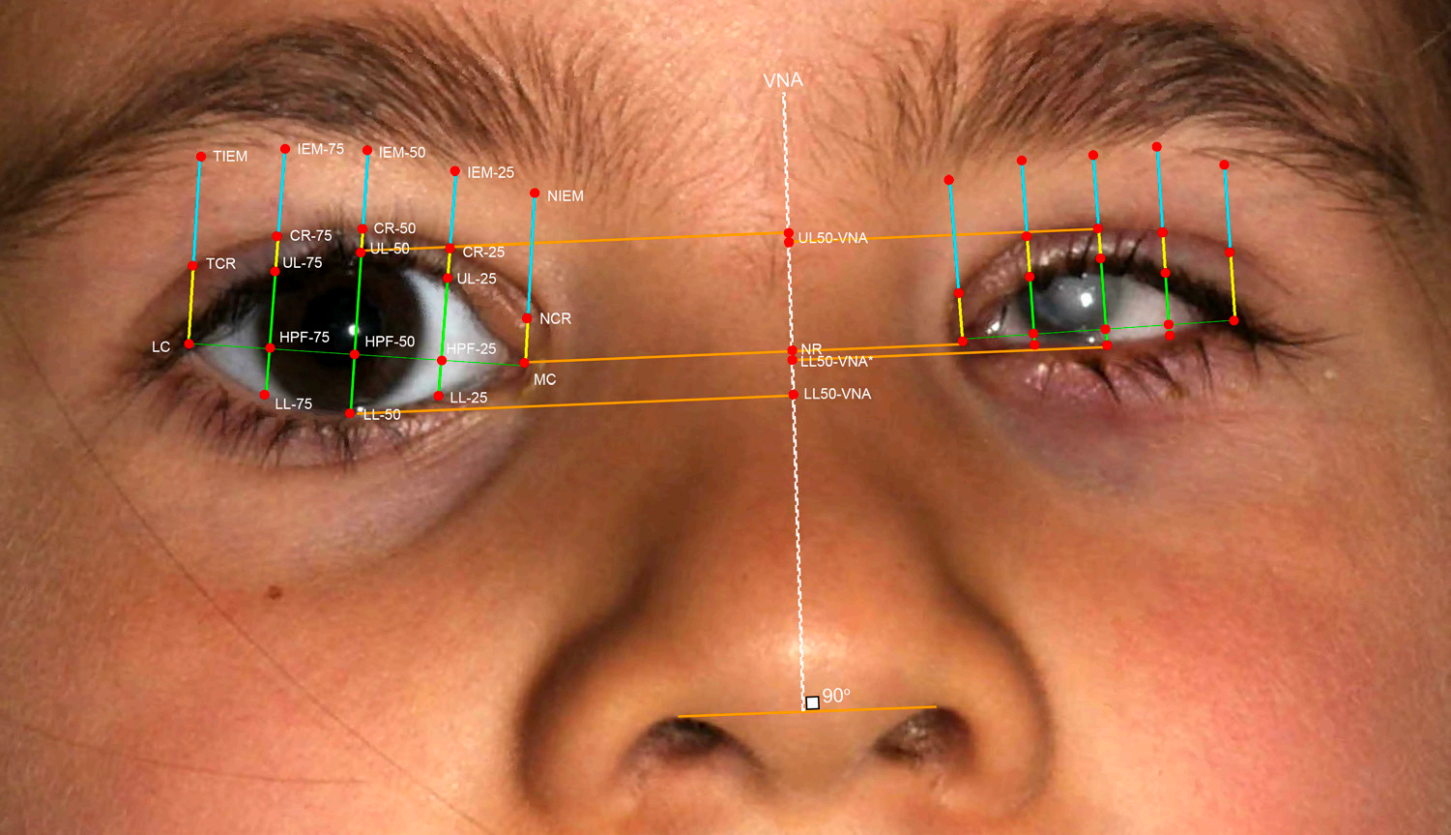
Outline of the facial landmarks: medial canthus (MC) and lateral canthus (LC) were manually marked and connected. The line was evenly divided into 4 quarters and set at 0, 25, 50, 75, and 100 % of the total distance. Perpendicular lines were drawn through these points to set landmarks at the lower lid (LL), upper Eyelid (UL), lid crease (CR), and inferior eyebrow margin (IEM). HPF, horizontal palpebral fissure; NCR, nasal lid crease; NEM, nasal eyebrow margin; TEM, temporal eyebrow margin.

We also calculated caudal displacement of the eye by using the difference in coordinates on the craniocaudal axis. Enophthalmos was calculated by averaging the anteroposterior coordinates of the central eyelids (UL50 and lower eyelid 50). The difference in coordinates between OU was used. We choose to measure enophthalmos using the eyelid position, rather than the bulbus position, since measurements of the cornea are inaccurate due to the lack of capturing it with the 3D scanner.

### Orbital Measurements

Orbital measurements were performed by one radiologist (P.de.G.) using transpalpebral ultrasound using different machines (Toshiba Aplio 500, Toshiba Aplio i700, Aloka Prosound F75 and Philips EPIC 5) and high-frequency linear array transducers (range 14–18 MHz), and included orbital rim width defined as the maximal distance from lateral orbital rim to nasal maxilla), orbital rim height; maximal distance from anterior rim of orbital floor inferior to frontal bone superior), and AL; anterior cornea to macula. In the case of anophthalmia, without a visible structure on ultrasound, the AL was set as 0 mm. Relative values were calculated by dividing the affected orbit and eye measurements by the unaffected measurements.

### Subjective Judgment

Subjective satisfaction score was judged from a combined set of 2D and 3D facial images. It was performed by 4 professionals: an ocularist (J.S.R.), an orbital reconstructive surgeon (D.T.H.), an ophthalmologist (A.L.W.G.), and a clinical researcher (E.J.R.). Scores were given on the overall appearance of the face, where the judges were asked to give a judgment with the ocular prosthesis in place, using a Likert-scale from 1 to 5, where 1 was denoted as mild, 2 as mild-moderate, 3 as moderate, 4 as moderate-severe, and 5 as severe outcome. The average scores of the 4 observers were used as a study parameter.

### Analysis

Statistical analysis was done in SPSS 28. The rHPF was checked for interobserver (3 independent observers) correlation using Cronbach’s alpha to test for reproducibility of the 3D measurements. Data were tested for normality using a Q-Q plot. The correlation between subjective outcome as dependent variable and orbital size, AL, and the facial measurements was analyzed with a bivariate linear regression model. The score of the 4 observers was first checked for interobserver correlation using Cronbach’s alpha. In total, 10 ultrasound and facial parameters were compared with the subjective outcome. To reduce the risk of a type-1 error, the Bonferroni correction was used. This results in a significance level alpha of 0.005 (0.05/10 = 0.005). After the bivariate linear regression models, a forward selection procedure was performed to identify the most influential associations with the subjective outcome. The criteria for variable entry and removal were set at a probability of F-to-enter ≤0.05 and a probability of F-to-remove ≥0.10. Axial eye length was excluded from the multiple regression model since this parameter is not influenced by treatment.

A correlation matrix between all variables, which were significantly associated with the subjective outcome, was made. Moderate correlation was defined as r between 0.3 and 0.7, and high correlation was defined as r >0.7.

## RESULTS

Thirty-one patients with a unilateral microphthalmia or anophthalmia were included. Patient characteristics and subjective scores are shown in Table 1. Twenty-seven patients had microphthalmia, and 4 patients were anophthalmic. rHPF had a strong interclass correlation coefficient between the different observers with a Cronbach’s alpha of 0.92. Subjective judgment also had a strong interclass correlation coefficient between the different observers, with a Cronbach’s alpha of 0.94.

**TABLE 1.** Characteristics of the population categorized by subjective scores

Outcome	Subjective score 1-<2 Excellent	Subjective score 2-<3 Good	Subjective score 3-<4 Fair	Subjective score 4-5 Poor
Number of patients	16	6	4	5
Side-affected eye	R6/L10	R4/L2	R3/L1	R4/L1
Age in years (at time of scan)	5.4 ± 3.61	3 ± 0.63	7.3 ± 3.69	8.8 ± 5.22
Subjective judgment	1.2 ± 0.3	2.4 ± 0.31	3.4 ± 0.14	4.6 ± 0.41
HPF affected eye	21.4 ± 1.97	20 ± 2.14	20 ± 0.95	17.9 ± 2.22
HPF normal eye	22.7 ± 1.61	21.8 ± 0.36	24.1 ± 1.41	24 ± 1.39
rHPF	0.9 ± 0.1	0.9 ± 0.1	0.8 ± 0.05	0.7 ± 0.07
Axial length affected eye	13.4 ± 4.84	8.1 ± 3.21	10.6 ± 1.34	4.1 ± 6.6
Axial length normal eye	22.2 ± 1.49	21.1 ± 0.82	23.6 ± 1.7	21.8 ± 1.84
Relative Axial length	0.6 ± 0.26	0.3 ± 0.23	0.2 ± 0.28	0.2 ± 0.43
Orbit width affected eye	30.9 ± 1.75	28.4 ± 1.36	30.9 ± 0.54	26.3 ± 1.12
Orbit width normal eye	33.9 ± 1.46	33 ± 1.07	33.5 ± 1.87	35.9 ± 1.42
Relative orbital width	0.9 ± 0.06	0.9 ± 0.05	0.9 ± 0.03	0.7 ± 0.05
Orbit height affected eye	28.6 ± 2.74	27.1 ± 1.95	29.7 ± 3.62	25.3 ± 1.76
Orbit height normal eye	32.4 ± 1.79	31.5 ± 1.11	33.8 ± 1.95	32.9 ± 1.95
Relative orbital height	0.9 ± 0.09	0.9 ± 0.06	0.9 ± 0.07	0.8 ± 0.1
Brow height affected eye	11.6 ± 2.54	12.2 ± 3.5	12.6 ± 1	10.1 ± 1.77
Brow height normal eye	11.6 ± 3.31	11.4 ± 2.81	12.9 ± 2	12.2 ± 2.69
Relative brow height	1 ± 0.16	1.1 ± 0.24	1 ± 0.11	0.8 ± 0.13
VPF affected eye	7.6 ± 2.64	8 ± 1.4	6.8 ± 1.16	8.1 ± 0.54
VPF normal eye	8.3 ± 1.81	7.9 ± 2.18	9.5 ± 1.93	9.4 ± 1.25
rVPF	0.9 ± 0.19	1.1 ± 0.47	0.7 ± 0.06	0.9 ± 0.13
Caudal placement	0.2 ± 1.62	1.4 ± 1.23	3.8 ± 1.96	4.1 ± 3.45
Ratio HPF/VPF affected eye	0.6 ± 1.51	1.7 ± 1.56	3.5 ± 2.35	3.6 ± 2.71
Ratio normal eye	3.2 ± 1.17	2.6 ± 0.38	3 ± 0.62	2.2 ± 0.29
Enophthalmos	2.9 ± 0.72	3.1 ± 1.42	2.6 ± 0.51	2.6 ± 0.28
Pretarsal show affected	1.1 ± 0.21	0.9 ± 0.25	1.2 ± 0.15	0.9 ± 0.11
Pretarsal show unaffected	0.8 ± 2.06	0.1 ± 2.06	0.5 ± 0.59	1.2 ± 2.77
Pretarsal show %	3.6 ± 1.29	4 ± 1.73	5 ± 1.45	4.7 ± 0.89

Absolute measurements are in mm. Relative values are shown in decimals (mean ± standard deviation).

rHPF, relative horizontal palpebral fissure; r ± VPF, relative vertical palpebral fissure.

Overall subjective judgment showed a significant correlation with rHPF (*p* < 0.001), caudal displacement of the eye (*p* < 0.001), orbital width (OW) (*p* = 0.001), pretarsal show (*p* = 0.003), and AL (*p* = 0.003) (Table 2).

**TABLE 2.** Bivariate regression analysis for subjective scores

Subjective score	Unstandardized B (95% CI)	R square	*p* value
Orbit width	9.21 (3.99–4.43)	0.32	0.001*
Orbit height	5.72 (0.17–11.27)	0.14	0.044
Axial length	2.14 (0.81–3.47)	0.28	0.003*
rHPF	7.95 (4.82–11.07)	0.48	<0.001*
VPF	2.33 (−0.58 to 5.25)	0.09	0.11
Ratio rHPF/rVPF	0.34 (−0.16 to 0.85)	0.06	0.17
Pretarsal show	−1.46 (−2.37 to −0.55)	0.29	0.003*
Brow height	1.89 (−0.53 to 4.30)	0.09	0.12
Caudal displacement	−0.31(−0.47 to −0.15)	0.36	<0.001*
Enophthalmos	−0.04 (−0.28 to 0.20)	0.00	0.72

* Correlation is significant at the 0.005 level; the alpha is corrected for repeated measurements using a Bonferroni correction.

rHPF, relative horizontal palpebral fissure; rVPF, relative vertical palpebral fissure.

In the first step of the forward selection procedure, rHPF was added to the model, resulting in a significant model. F (1,25) = 14.93, *p* < 0.001. The addition of the other significant results did not lead to a significant improvement of the model.

The correlations between all significant variables were then calculated (Table 3). A moderate, statistically significant correlation with OW was found for AL (0.55) and rHPF (0.60). rHPF also showed a moderate, statistically significant correlation with pretarsal show (−0.53) and caudal displacement (−0.47).

**TABLE 3.** Correlation matrix for all significant parameters

	Orbital width	Axial length	rHPF	Pretarsal show	Caudal displacement
Orbital width	1	0.55*	0.60*	−0.18	−0.17
Axial length	0.55*	1	0.42*	−0.11	−0.22
rHPF	0.60*	0.42*	1	−0.53*	−0.47*
Pretarsal show	−0.18	−0.11	−0.53*	1	0.39*
Caudal displacement	−0.17	−0.22	−0.47*	0.39*	1

* Correlation is significant at the 0.05 level.

rHPF, relative horizontal palpebral fissure.

## DISCUSSION

These results underscore the importance of rHPF as an objective outcome for MICA patients. Apart from rHPF, increased pretarsal show asymmetry, smaller OW, and increased caudal displacement were also associated with a lower clinical judgment, rHPF showed a strong multicollinearity with the other significant factors; however, the forward selection showed that rHPF has a better predictive value for the clinical judgment and may therefore be considered as the primary clinical outcome measure for MICA.

Orbital height, VPF, the ratio between rHPF/rVPF, brow height, and enophthalmos, did not correlate with subjective outcome.

Anatomically seen, the OW is likely to reflect the maximum HPF. One should be aware that the HPF might be smaller in case no conformer therapy has started from an early age because the eyelids are not fully stretched in case of the small or absent eye. In our cases, conformer therapy was started from an early age, which likely explains the high correlation in our population.

The pretarsal show symmetry also showed an association with subjective judgment, where a more symmetric pretarsal show results in a better score. However, it should be noted that it is again strongly correlated with rHPF and is not considered an independent factor. Nevertheless, care should be taken to minimize the difference in pretarsal show, such as ptosis repair, lid crease reconstruction, and adding sufficient volume in the socket or preaponeurotic part of the orbit.^22^

In the bivariate analyses, we saw that caudal displacement was associated with a lower score. This measurement is, however, also correlated with the rHPF and therefore not considered to be an independent variable. As shown by the study of Yuan et al.^23^ the bony part will rather be higher on the affected side. The caudal displacement seen in our examples is therefore likely due to gravitational forces from a heavy prosthesis causing lowering of the eyelids or a lower position due to an inadequate fornix inferior. A potential solution might be surgical (fornix deepening procedure or socket reconstruction) or directed conformer therapy aiming to deepen the fornix inferior.^24,25^

rHPF is a significant and easily measured predictor, as it can be done with a simple 2D or 3D photo or even a handheld ruler. This parameter provides a representation of subjective judgment and is suggested as the main decision tool and clinical outcome measurement in microphthalmic and anophthalmic patients. Ultrasonography will, however, add information on anatomy, AL, follow-up of a potential cyst, and change in bony dimensions, which will add information for the individual treatment choices.

In this study, we used 3D scanning to compare multiple parameters and dimensions combined in one measurement. However, since rHPF, pretarsal show, and caudal displacement are all parameters that can be measured in the same (frontal) plane, 2D photographs will likely be sufficient. Earlier studies showed that the results between 2D and 3D imaging in the periocular region were comparable.^26^ For better interpretation, further quantification between 2D and 3D can be applied.

The AL is another factor that correlates with the subjective score in the bivariate analysis, where a smaller eye results in worse outcome. The AL also correlates with the OW and the HPF. The correlation between AL and HPF was also seen in the pretreatment phase in an earlier study,^18^ and the HPF was also correlated to orbital volume in untreated patients in the study of Jiang et al.^27^ Although the AL is a predictor for clinical outcome, it is not regarded as an outcome measure, and therefore not included in the multivariate analysis.

With this study, we showed various objective measurements that associate with subjective outcome in MICA, where rHPF is the best predictor for outcome and is also directly correlated with the OW. The pretarsal show and caudal displacement are other potential treatable factors that can improve the facial appearance of these children. To our best knowledge, this is the first study evaluating objective outcome measurements by comparing facial measurements with orbital measurements as a way to track treatment outcome.

It should be noted that the current study only focuses on the search for an outcome measurement that is correlated with subjective judgment and can now be used as an easily accessible objective outcome parameter, for example, to be able to compare grouped results internationally, or if guidelines are considered for treating these children. Since the measurement also reflects the bony dimensions, there is no need to include invasive CT scans for follow-up. For sure, there are other outcomes that play a role in the definition of success, like the pretarsal symmetry, and the caudal displacement of the artificial eye (shown in this study), but also factors like a normal blinking capacity, and most importantly, the comfort of the child. For individual patient care, the HPF outcome measurement may be used to guide the individual-based treatment plan. For example, when sufficient symmetry of the HPF has been achieved, the frequency of prosthetic exchanges can be decreased. It is, however, important to note that MICA is a complicated disorder with different grades of severity and potentially other physical or psychological factors, where the well-being of the child should always be the primary target. The choice to treat, the treatment plan, and the aimed outcome are carefully based on the individual situation. The aim should not simply be to achieve full symmetry of the HPF, but a treatment plan should be targeted on reasonable expectations with good care for the comfort and safety of the child.

In conclusion, it is indicated that the HPF is the best predictive factor for subjective outcome. Followed by caudal placement of the eye, OW, pretarsal show, and AL. It should be noted that the HPF and OW have a high multicollinearity, where HPF has a better correlation with the subjective outcome compared with OW.
